# Label-Free Determination of the Number of Biomolecules Attached to Cells by Measurement of the Cell's Electrophoretic Mobility in a Microchannel

**DOI:** 10.1371/journal.pone.0015641

**Published:** 2010-12-29

**Authors:** Atsushi Aki, Baiju G. Nair, Hisao Morimoto, D. Sakthi Kumar, Toru Maekawa

**Affiliations:** Bio-Nano Electronics Research Centre, Toyo University, Kawagoe, Japan; University of California, Berkeley, United States of America

## Abstract

We developed a label-free method for a determination of the number of biomolecules attached to individual cells by measuring the electrophoretic mobility of the cells in a microchannel. The surface of a biological cell, which is dispersed in aqueous solution, is normally electrically charged and the charge quantity at the cell's surface is slightly changed once antibody molecules are attached to the cell, based on which we detect the attachment of antibody molecules to the surface of individual red blood cells by electrophoretic mobility measurement. We also analyzed the number of antibody molecules attached to the cell's surface using a flow cytometer. We found that there is a clear correlation between the number of antibody molecules attached to the individual cells and the electophoretic mobility of the cells. The present technique may well be utilized not only in the field of cell biology but also in the medical and pharmaceutical industries.

## Introduction

Numerous types of proteins such as ion channels, receptors and antigens are embedded in the membranes of biological cells and some regions of those proteins appear outside the cells' surfaces. Those proteins are interacting with other foreign biomolecules and ions under different physiological conditions [1]. The activities of all the organisms such as electric signal transfer, ATP synthesis and cells' adhesion are controlled by the biochemical interactions occurring at the surfaces of living cells [2], [3], [4]. Investigating the biochemical events occurring at the surfaces of the cells is important in the fields of cell biology and biochemistry and as a result, a number of different techniques for the estimation of the interactions between biomolecules and the membranes of cells have been developed [5], [6], [7]. Baksh et al. presented a simple protein-binding assay that utilizes the structural change in clusters composed of microparticles derivatized with lipid-membranes, which is induced by the attachment of proteins to the membranes on the particles [5]. Detecting antigen-antibody reactions occurring at the surfaces of living cells is essential for investigating the membrane structures of individual living cells and therefore has been carried out in the field of cancer, human immunodeficiency virus (HIV) and malaria diagnosis [8], [9], [10], [11], [12]. For instance, Nagrath et al. demonstrated a capture of circulating tumor cells (CTCs), which would be causing metastasis of cancer to the other parts of a body, onto the surfaces of micropillars modified with the antibody molecules against the CTCs in a microchip, using a cancer patient's whole blood [8].

In order to detect the antigen-antibody reactions at the surfaces of biological cells, the antibody molecules modified with fluorescent dyes are often attached to the cells and the fluorescence intensity of the dyes is measured using a fluorescent microscope, spectrometer or flow cytometer, through which a number of new findings and ideas have been derived in the field of life science [13], [14], [15]. However, the above facilities and equipment are, in general, large-scale and expensive due to complicated optical components such as light sources, photomultipliers, wavelength filters etc. Advanced synthesizing techniques are also required for the fluorescence labeling onto antibody molecules. In the case of cellular analysis using monoclonal antibodies in particular, there is an urgent demand for label-free detections of antigen-antibody reactions at the surfaces of living biological cells.

When biological cells are dispersed in aqueous solution, electric double layers are established around them since the surfaces of the cells are normally electrically charged. If an electric field is applied to the cells' suspension, the cells move in the direction of the electric field. Note that the electrophoretic mobility of each cell is proportional to the charge quantity at the cell's surface. Once antibody molecules are attached to the surfaces of the cells, the surface charges are slightly changed and as a result, the electrophoretic mobilities alter [16], [17]. Utilizing this phenomenon, antigen-antibody reactions at the cells' surfaces have been detected without any labeling onto the antibody molecules in a microchannel [18], [19]. However, quantitative label-free analysis of the number of antibody molecules attached to the surface of each cell has not yet been carried out using a microdevice. In this article, we present a label-free method for a determination of the number of biomolecules attached to individual cells by measuring the electrophoretic mobility of the cells in a microchannel.

## Materials and Methods

**Figure 1:**
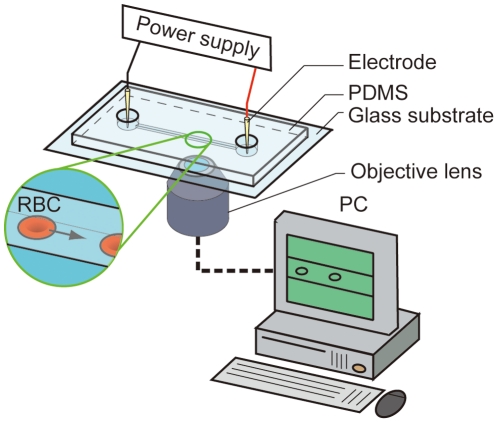
Schematic diagram of an electrophoresis experimental system. Two conical electrodes are set at both the inlet and outlet of a microchannel. Cells are moved along the microchannel by applying a dc electric field.

The outline of the electrophoretic mobility measurement system is shown in Fig. 1. We fabricated a microchannel on the surface of a polydimethlsiloxane (PDMS) substrate by the conventional soft lithography method [20]. First, we made a micropattern on SU-8 2035 (MicroChem, MA) attached to the surface of an Si substrate with the UV lithography method, poured PDMS liquid (Momentive, NY) into the micropatterned substrate and left it at rest for 12 h at room temperature in order to solidify the PDMS liquid. We peeled the solidified PDMS substrate from the micropatterned Si substrate, made two holes at the ends of the microchannel and attached the PDMS substrate to the glass substrate. The length, width and height of the microchannel were 10 mm, 20 µm and 35 µm, respectively. In order to coat the surface of the microchannel with 2-methacryloyloxyethylphosphorylcholine (MPC) molecules, we dissolved Lipidure CM5206E (NOF, Japan) in water, injected the MPC solution into the microchannel and incubated the microchannel at 40°C for 12 h. Thanks to the MPC coating, the surface charge of the microchannel was remarkably reduced and the cells' adhering on the surface of the microchannel was prevented. We suspended sheep's whole blood (Nippon Bio-Supp. Center, Japan) in phosphate buffered saline (PBS) of pH 7.0 and centrifuged the PBS/blood solution at 2500 rpm for 5 minutes. Then, we removed the supernatant liquid from the suspension and added the same amount of PBS to the suspension. We repeated the above procedure four times in order to suspend only RBCs in PBS, removing the other components such as leukocytes, platelets and plasma. The hematocrit was 0.5%. Anti-sheep's RBC IgG (Rockland, PA) was added to the RBC suspension and they were mixed together at room temperature for 1.5 h. In the case of the flow cytometry experiment, the above IgG molecules were labeled with fluorescein isothiocyanate (FITC) molecules. We centrifuged the RBC suspension and replaced the supernatant solution of the RBC suspension with PBS in order to remove the excess amount of IgG molecules. We then carried out flow cytometry and electrophoresis experiments. First, we counted the number of IgG molecules attached to the surfaces of the individual RBCs using a flow cytometer (BD FACS Calibur; Beckton, Dicknson and Company, NJ). We established a calibration curve in order to find the correlation between the fluorescence intensity and the number of fluorescent dye molecules at the surfaces of microparticles. We measured the fluorescence intensity of four populations of reference particles (Quantum FITC MESF kit low level; Bangs Laboratories, Inc., IN) using the flow cytometer. There was a linear correlation between the peak fluorescence intensity of each population and the value of molecular equivalent soluble fluorochrome (MESF), which is proportional to the number of fluorescent dye molecules at the surface of each particle. Then, we measured the fluorescence intensity of RBCs, which had reacted with IgG molecules attached to FITC molecules, and estimated the value of the MESF of the RBCs. The concentration of IgG was changed as follows; 1.5, 3.0, 15 and 30 µg ml^−1^. Since each IgG molecule was combined with two FITC molecules on average, the actual number of IgG molecules attached to the RBCs was one-half of the MESF value.

We also measured the electrophoretic mobility of Human U251 Glioblastoma cells (JCRB, Japan) in order to see if the surface charge also alters after the attachement of the antibody molecules to the cancer cells. Cells were cultured as exponentially growing monolayers on 25 cm^2^ (surface area) of flask in EMEM medium (GIBCO, CA) supplemented with 10% (v/v) fetal bovine serum and 1% antibiotic solution. For experimental studies, cells were harvested using scrappers and cell dissociation buffer without any trypsin treatments. After harvesting, the number of cells was counted using Countess™ Automated Cell Counter (Invitrogen, CA) and maintained at 10^6^ cells ml^−1^. All of the cells were dispersed in the fresh PBS and mixed with the antibody, anti-IL13RA2, the concentration of which was 25 µm ml^−1^. Finally, we injected the cells into the microchannel for the electrophoretic mobility measurement.

## Results

**Figure 2:**
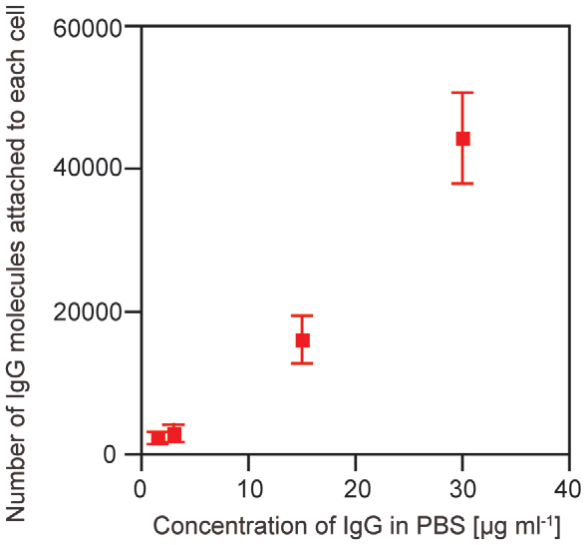
Dependence of the number of IgG molecules attached to the surface of a RBC on the concentration of IgG molecules in PBS. The number of the IgG molecules was measured by a flow cytometer.

The number of IgG molecules attached to the surface of each RBC is shown in Fig. 2. The number increased almost linearly with an increase in the concentration of IgG molecules. Secondly, we measured the electrophoretic mobility of RBCs. We injected a small amount of the RBC suspension into the microchannel and applied a dc electric field of 2.2×10^3^ V m^−1^. The movement of the individual RBCs in the microchannel was observed by an inverted microscope, recorded on a hard disk and analyzed by a computer. We measured the electrophoretic mobility of 100 RBCs without any surface reactions, the result of which agreed well with that obtained by a conventional machine (ELS-6000; Otsuka Electronics, Japan) [18]. We also measured the electrophoretic mobility of 100 RBCs, which had reacted with IgG molecules, in the microchannel. The absolute value of the electrophoretic mobility of the RBCs that had reacted with IgG molecules decreased compared to that of the RBCs without any reactions. There is a clear correlation between the electrophoretic mobility of the RBCs and the concentration of IgG molecules; that is, the absolute value of the surface charge of the RBCs decreased after the attachment of antibody molecules to them [19]. The attachment of antibody molecules to the surfaces of individual RBCs was successfully detected by measuring the electrophoretic mobility even when the concentration of the antibodies was much lower than that employed by the conventional agglutination method.

**Figure 3:**
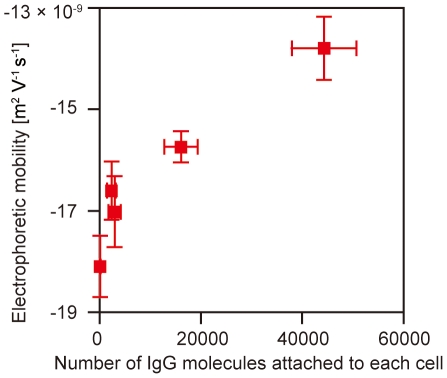
Correlation between the electrophoretic mobility of each RBC and the number of IgG molecules attached to the cell's surface.

Finally, we combined the above result obtained by the electrophoretic mobility measurement, that is the relation between the electrophoretic mobility of individual RBCs and the concentration of IgG molecules in the PBS, with the first result obtained by the flow cytometry experiment, that is the relation between the number of IgG molecules attached to the RBCs and the concentration of IgG molecules in the PBS, the result of which is shown in Fig. 3. It is clear that there is a correlation between the electrophoretic mobility of the RBCs and the number of IgG molecules attached to the surfaces of the RBCs. In other words, we can estimate the number of antibody molecules attached to the surface of each cell by measuring the electrophoretic mobility of each cell in a microchannel.

We also cultivated Human Glioblastoma cells, and measured the electrophoretic mobility of the cells which had been reacted with anti-IL13RA2. The electrophoretic mobilities of the cells, to which the antibody molecules were attached, was −4.08±0.14 m^2^ V^−1^ s^−1^, whereas that of the cells without any reaction with the antibody molecules was -4.18±0.39 m^2^ V^−1^ s^−1^. We found that the average electrophoretic mobility of the cells slightly decreased by attaching the anti-IL13RA molecules to the antigen around the cell's surface. The actual number of the antibody molecules attached to the cancer cells can be estimated in the same way as in the case of RBCs.

## Discussion

We demonstrated that a label-free determination of the number of biomolecules attached to individual cells is possible by measuring the electrophoretic mobility of the cells in a microchannel. We suppose that the electrophoretic mobilities of different cells change in general after the attachment of the antibody molecules to the surfaces of the cells and the number of the antibody molecules can be estimated by measuring the electrophoretic mobility in a microchannel. The present technique does not require any labeling molecules such as enzymes, fluorescent dyes or radioisotopes. Therefore, this method may well make a great contribution to progress in the investigation of membrane structures using, for instance, self-produced monoclonal antibodies so that specific rare cells can be identified. What is more, the present system has great potential in carrying out high-throughput analysis of the surface structures and biochemical reactions of living cells. The electrophoretic mobility measurement shown here can be automated by incorporating cell detectors such as optical fibers or a Coulter counter [21] into the microchannel system, thanks to which the velocity of a large number of living cells can be quickly measured in a microchannel [22], [23], [24]. We are convinced that the present system can be utilized not only in the field of cell biology, but also in the medical and pharmaceutical industries.
